# How to Plan Research in Palliative Care

**DOI:** 10.4103/0973-1075.76230

**Published:** 2011-01

**Authors:** Santosh K Chaturvedi

**Affiliations:** National Institute of Mental Health & Neurosciences, Bangalore, India

**Keywords:** Palliative care, Planning, India

## Abstract

Research in palliative care has its challenges. However, research in different aspects of palliative care is important. This paper gives simple methods of planning and conducting a research in the area of palliative care in India.

## INTRODUCTION

Research is important in any area of science, health, and medicine to help professionals find new observations, insights, understandings, and treatments. Palliative care is a relatively new field, especially in India, and requires much active researches to give us knowledge based on local settings and environment.

The research or study may be done as an academic requirement for the educational course as a thesis or dissertation, or for individual interest, or through funding opportunity. It may be done for making a presentation at a conference and overall for career growth.

The research should be a systematic investigation or evaluation, done with a clear purpose or objective, based on observable experience in an unbiased way, leading to the possible or probable answer to the investigative question. The research should be such that is of benefit or definite implications like either adding to existing knowledge, providing better understanding of a phenomenon, or a new finding or intervention.

## TOPICS FOR RESEARCH

This should begin with writing and planning a research protocol. The first step is identifying the area on which a researcher wants to do a study. In palliative care, common broad areas can be related to:

Pain and pain relief

Other symptoms like fatigue, lymphoedema, delirium, nausea, cachexia, etc

Psychological issues like depression, demoralization, phobias, anxiety, panic

Social factors like social stresses, social support, family issues

Communication issues like collusion, difficult questions

Interventions-pharmacological or non-pharmacological, complementary methods

Staff stress and burnout

Systematic reviews and meta analysis

Grief and bereavement

Once the area is identified, the researcher should ask a research question to himself or herself. This would form the aim/s of the research. This is perhaps the most important initial decision to be made, as once this is decided, the objectives, hypotheses, and methodology can be planned. This should arise as a curiosity for the researcher.

## REVIEW OF LITERATURE

There are two main reasons for this. First, to know about what is already known about the theme one wants to pursue? Second, to know about other studies that had pursued the area, and what were their limitations, and plan a study overcoming those limitations. The literature search should be thorough, after consulting relevant books, journals, internet sources and search engines. The Medline/PubMed, Google Scholar, Scopus, Indian Medlars, Directory of open access journals, and many other are easily available to help in the search. It is useful to discuss with the supervisor, guide or someone who has already done similar work.

## SETTING AN AIM

This could be a question or a statement. If this is a question, it could be like-What is the severity of cancer pain at the time of admission into a hospice or palliative care setting; what is the nature and prevalence of anxiety, worries, depression, demoralization, in a palliative care centre; how does grief manifest among the relatives of the cancer patient; when does the nausea increase or decrease; how to measure fatigue in terminal stages; why does one get staff stress and burnout. The research question could be-what, why, when, where, why, how, how much, etc.

When the aim is a statement, it could be like-to study the clinical and demographic factors related to terminal delirium or to study the efficacy of newer analgesic in the management of cancer pain, to understand the development of stress among palliative care professionals. The aim should be as specific as possible, which would make it easier to plan out an appropriate methodology for the study.

The objectives basically split the aims into specific parts of the main aim. For example, on a study to assess prevalence of sleep disturbances in those with advanced disease, the objectives could be laid down as-to determine the occurrence of sleep disturbances in those with advanced disease, to correlate the sleep disturbance with different clinical and demographic factors, and to study the characteristics/nature of sleep disturbances.

## PLANNING THE METHODOLOGY

The methodology should provide the way to seek the answer to the question set out as aim. One needs to think over, the method to get an answer or information for the main aims and objectives laid down. Think of ways in which the answer to the research question could be sought and the advantages or limitations of each method. If needed, a Pilot phase of the study can be conducted to evaluate the feasibility of the study, time taken per subject, and any difficulties encountered.

The research methodology indicates the type of the study. The studies could be

### Qualitative or quantitative

Qualitative studies look at detailed accounts of an experience or phenomenon, and may ask broad, open ended, interconnected questions, the answers of which give insights into the event, rather than counting number of subjects with the experience. These are based on detailed interviews, focus groups, case studies, guided interviews, and knowledge attitude behavior patterns studies. The results are in the form of transcripts and verbatim accounts. Qualitative methods are often used in unstudied or understudied areas and may lead to a quantitative study and vice versa.

Quantitative studies are the traditional studies with a number of subjects which are analyzed to give patterns of differences in numbers, rather than actual experiences.

### Descriptive or comparative

Descriptive studies tend to examine the features of a phenomenon and its description, whereas the comparative study compares these observations with another group of patients with similar problems or a normal healthy control group.

### Retrospective or prospective or cross-sectional study

A *cross-sectional* study provides information about the situation that exists at a single point of time. These could be on disease or symptom description or process. A longitudinal study provides data about events or changes during a period of time, it may be *retrospective*-if previously recorded data or observations made before the start of study is used, or *prospective*- if data is recorded or observations are made after the start of study. Prospective and retrospective studies may be on an intervention or on observations.

### Open studies or blind studies

In open studies, the subjects and researchers are aware of the intervention and who is getting it. In single blind study, either the subject or the researcher is not aware of the intervention; in double blind, both the subject and the rater are unaware about the intervention and who is getting it or not. Blinding is done to remove rater or subject bias.

Randomized controlled trials or double blind placebo controlled trials are considered a stringent method as it removes many biases. The subjects are selected using random methods, and the study has a control group of other intervention or a placebo, and both the subject and rater are unaware of the intervention details.

Follow up studies are when a group of subjects are called back to study any changes in their condition after a period of time.

Cohort studies include a group of subjects who are observed periodically till the end of the study.

Case controlled studies are studies done where each case is compared with a control subject of similar characteristics.

## SAMPLE AND SAMPLING

The groups of individuals who are studied form the sample, and the procedure of selecting the sample is called sampling. Depending on the nature of study and the aims and objectives, the sample size needs to be decided. The source of sample also needs to be identified, whether the sample would be from general population, out patients, admitted patients, hospice, community care centre, from staff, relatives or caregivers. The study period also needs to be fixed in terms of days, weeks or months, or any prespecified period. The sample should be adequate, representative, and unbiased.

The inclusion and exclusion criteria can be fixed to study the sample which needs to be studied and exclude unwanted or undesirable sample.

Sample size needs to be adequate for the study and varies between the types of the studies. It may be a few persons in a qualitative study or a large population depending on whether it is a descriptive study or intervention related. There are many ways of calculating the sample size, and the power of sample size can be derived which would make the findings reliable and valid. It is better to consult a statistician to do the power calculation and get advice on the adequate and appropriate sample size.

Identify variables to be studied. These should be related to the aims and objectives of the study. There may be a need to develop operational guidelines.

Scales or instruments or measures: Identify scales or instruments which will be helpful in seeking the answer to the research question. Choose standard measures, which are reliable, valid and have good psychometric properties, and preferably those which have been translated, adapted, and used in the Indian setting.

## PROCEDURE AND DATA COLLECTION

The procedure needs to finalized before stating data collection. This should start with seeking informed consent and explaining the purpose of the study. Information should be collected in an unbiased way. Once the subjects have been identified by using inclusion/exclusion criteria the data can be collected and recorded systematically. If an intervention is involved, this should be done in a standard way.

### Ethical aspects

Two ethical aspects which are key to any study are an approval of approval from the Institute Review board or ethics committee, and seeking informed consent from the subject and/or the relative, as the case may be. Nobody who does not consent need be included in the study.

### Statistical analysis

The data needs to be cleaned for inconsistency, inaccuracies and missing data handling. The data can be entered into a statistical package like SPSS or on an excel sheet. The statistics could be simple description of subjects with percentages, mean, median, standard deviation. If comparisons are made comparative statistics like chi-square or *t*-test can be used. Multivariate and other relevant statistics can be employed in discussion with the statistician. The levels of statistical significances or lack of this should be clearly mentioned. Common statistical tests, which may come in use are chi square, Fishers exact, odds ratio, Student’s *t*-test, ANOVA/MANOVA, correlation coefficients, regression, factor analysis, cluster analysis, and many more.

## RESULTS

These should be presented in neat simple tables which are self-explanatory and the important observations should be written in text as well.

## DISCUSSION

The main observations need to be discussed. Are the findings comparable to those of other similar studies? if so, how? If not, why not? The discussion should provide an interpretation of the findings.

### Limitations

There is no harm in acknowledging the limitations of the study, as all studies are bound to have some limitation or the other.

### Future directions

It is a good idea to give suggestions for overcoming limitations of the study, and indicate how the findings are heuristic and lead to further studies.

## CONCLUSION

The main observations should clearly indicate the conclusion, which is like the final answer to the question raised as the aim, even if, the answer is negative, incomplete, or inconclusive.

### Summary

A summary with the statement of aim, methods, main findings, interpretation, and conclusion makes a easy source for a reader to decide whether the complete report needs to be read or not.

## REFERENCES

Follow the standard format of writing references in the text and in the end in the list of references. These are usually dictated by the Institute or the journal. The references should be complete, and one should cross-check their accuracy.

Writing up thesis, dissertation, report, or paper: This is the final outcome of the study. The study completion may end up as a bound thesis or dissertation, or submitting the paper for publication in a peer reviewed journal. The findings can also be presented at any scientific conference.

Other tips: Always have a written research protocol which should be followed strictly without any variation. It is good to have views and suggestions of peer, teachers, and supervisors, which might help in overcoming any blind spots in the researchers. Research done with interest and passion is always a joy!

